# sA population-based study of macular choroidal neovascularization using optical coherence tomography in Eastern China

**DOI:** 10.3892/etm.2014.1731

**Published:** 2014-05-26

**Authors:** JIE ZHAO, JUN HU, HAO LU, LEI YANG

**Affiliations:** Department of Ophthalmology, Baoshan Central Hospital, Shanghai 201906, P.R. China

**Keywords:** age-related macular degeneration, choroidal neovascularization, optic coherence tomography, population-based study, Eastern China

## Abstract

The aim of the present study was to investigate the pathomorphological and functional variations of choroidal neovascularization (CNV) in age-related macular degeneration (AMD) in a Chinese population using optical coherence tomography (OCT). This population-based study enrolled 59 patients (age, >45 years; eyes, 70) with early and intermediate-stage AMD from Youyi Road Community, Baoshan District, Shanghai, China. Comprehensive standardized ophthalmic examinations included visual acuity, anterior segment analysis using a slit lamp, dilated fundus evaluation by direct ophthalmoscopy, 90D handheld lens analysis, fundus photography, fundus fluorescein angiography (FFA) and fast optic disk scans using OCT. The macular CNV characteristic profiles in early and intermediate-stage AMD were determined by OCT. Data were obtained on the first visit and the follow-up period ranged between 6 and 24 months, where FFA and OCT outcomes of early and intermediate-stage AMD patients were analyzed. Three profiles of early and intermediate-stage AMD were created from the OCT and FFA results, each with a different prognosis. Firstly, drusens with unclear boundaries and evident pigment proliferation, as well as hypofluorescence around the drusens, was observed via FFA. A slight small arch field located in the retinal pigment epithelium (RPE)/choriocapillary layer (CCL) was shown on OCT scans, indicating exudative AMD. Secondly, RPE detachments of >1 pupillary distance, without CNV in the macular area, indicated geographic chorioretinitis atrophy. Finally, drusens with clear boundaries and few pigment proliferations and no certain surrounding fluorescence was observed via FFA, while a clear RPE/CCL band on the OCT scans indicated slow progress. The results of the present study demonstrated that combined OCT and FFA was the most efficient method for identifying CNV and diagnosing AMD. If the two techniques are not available concurrently, then OCT is a safer and more reliable technique to follow-up early and intermediate-stage AMD patients.

## Introduction

Age-related macular degeneration (AMD) is a disease of the ocular fundus characterized by the degeneration of the choroidal capillaries, retinal pigment epithelium (RPE) and neural retina. AMD is the leading cause of blindness in older adults (>50 years-old) in Western countries (1). With living standards improving in China, the incidence of AMD is also increasing.

Traditionally, AMD may be classified into dry and wet forms by physicians. The dry form, or central geographic atrophy, accounts for 15–20% of AMD cases, where vision loss is ultimately caused by the loss of photoreceptors. Wet AMD, also known as neovascular or exudative AMD, results from choroidal neovascularization (CNV) and affects 80–85% of those with AMD. The wet form causes the more severe loss of vision. In the natural course of the disease, 10–20% of dry AMD cases become wet AMD cases, and 42% of patients with monocular wet AMD develop binocular wet AMD within five years (2).

Recently, a large-scale epidemiological study (n=100,000) undertaken in Shanghai First People’s Hospital (Shanghai, China) reported that the prevalence of AMD in individuals aged >45 years was 16%; wet AMD alone was 15% (3). AMD is the most common manifestation of CNV, and CNV usually leads to repeated macular bleeding, exudation and scarring, seriously damaging central visual acuity. As a result, investigation into the pathogenesis of AMD is urgently required and important for the prevention or delaying of AMD progression (4–7).

The present study included an epidemiological survey of ocular diseases in an adult population (>45 years old) in Youyi Road Community, Baoshan District, Shanghai, China, conducted to determine the prevalence of AMD in the region. For this purpose, routine ocular examinations were utilized, namely fundus photography, fundus fluorescein angiography (FFA) and ocular optical coherence tomography (OCT). Patients found to have early and intermediate-stage AMD were further recruited for follow-up observation. In these patients, the clinical and imaging characteristics of various stages of AMD were investigated, as well as the CNV characteristics of different etiologies. The present study aimed to further the understanding of the pathomorphological and functional variations of CNV in AMD patients, and optimize the guidance for the treatment of AMD.

## Materials and methods

### Subject recruitment

The study was a prospective, population-based study investigating the pathomorphological and functional variations of CNV in AMD patients in a Chinese population. In total, 1,813 adult citizens aged >45 years were recruited through probability sampling from Youyi Road Community, Baoshan District, Shanghai, China between April 2005 and June 2005 for an epidemiological survey. All the participants provided written informed consent. The study was approved by the local Ethics Committee of Shuguang Hospital (Baoshan branch, Shanghai, China) and was conducted in accordance with the Declaration of Helsinki.

### Ocular examination

All the participants underwent a preliminary assessment of near and far visual acuity, slit-lamp biomicroscopy and a dilated fundus examination with direct ophthalmoscopy and a 90D handheld lens. For those subjects with suspected early and mid-stage AMD, further ocular examinations, including fundus photography, FFA and fast macular map and line scans using OCT, were performed. The best corrected visual acuity was assessed monocularly with linear logMAR charts, if the subject had an uncorrected visual acuity of <1.0 (less than 20/20 vision). Pupillary dilation was induced with three cycles of 0.5% tropicamide (one drop per eye), administered 5 min apart. Fundus photography, FFA and OCT were performed synchronously using a Zeiss-Stratus OCT Model 3000 and an FF450 IR fundus camera (Carl Zeiss AG, Oberkochen, Germany).

According to the size and the number of drusens under the retina, dry AMD may be further classified into three stages: Early, intermediate and advanced stages (8). Dry AMD is the most common form of AMD in the early or intermediate stages. Early AMD was defined as the presence of coexisting multiple small drusens (diameter, <63 μm) and a few medium-sized drusens (63–124 μm) in the macular area. The presence of widespread medium-sized drusens and at least one drusen of >125 μm defined intermediate AMD. Advanced AMD was indicated by the presence of either geographic atrophy involving the fovea or exudative detachment induced by CNV. The subjects with early and intermediate-stage AMD were included in the present study. Subjects were excluded from the study if the FFA results were negative, fundus visibility was poor due to lens opacity or other reasons or if the patient had a history of central chorioretinitis or macular degeneration (which is difficult to distinguish from AMD). A trained technician performed all the scanning sessions.

### Follow-up

Subjects with early and intermediate-stage AMD were reviewed one year following the initial examination. Thereafter, the patients were reviewed for 6–24 months depending on the stage of AMD. Mean follow-up duration ranged from 18 to 24 months while the interval of follow-up of each patient ranged from 6–18 months. During the follow-up period, the following ocular examinations were performed: Best corrected visual acuity, anterior segment analysis by a slit-lamp, dilated fundus evaluation by direct ophthalmoscopy and a 90D handheld lens, fundus photography, FFA and OCT. Patients with angiogenesis or geographic atrophy were administered the appropriate drug treatment and were withdrawn from the study.

ICGA was performed using Carl Zeiss FF-450-IR Fundus Retinal Eye Medical Ophthalmic Camera System, 30° and 50° macular CF and indocyanine green ICG 25 mg (LiaoYang No. 3 Pharmaceutical factory, LiaoYang, China). After allergic test, rapid intravenous elbow injection of ICG was performed within 5 sec. ICGA observation duration was 30 min or more in total after injection in which the early stage was within 5 min, 10 to 20 min as a medium stage, and after 20 min as late stage of angiography. The results were analyzed by two ophthalmologists independently.

### Statistical analysis

All data are expressed as the mean ± standard error of the mean. Intergroup comparisons were conducted with the χ^2^ test using the SigmaStat-integrated SigmaPlot statistical software package (V 12.0; SPSS, Inc., Chicago, IL, USA). P<0.05 was considered to indicate a statistically significant difference for all the analyses.

## Results

### Patient characteristics

A total of 1,813 subjects were initially enrolled, however, only 1,619 individuals (89.3%) completed the study. Participants included workers with a number of professions, including manual labor, peasants, teachers and civil servants. The majority of the patients belonged to China’s main ethnic group (Han Chinese). The enrolled 1,813 subjects comprised 829 females (45.65%) and 987 males (54.35%), ranging in age between 45 and 82 years.

### AMD diagnosis

Among the 1,619 subjects who completed the study, 59 patients (3.64%, 70 eyes) were diagnosed as early and intermediate-stage AMD, while 42 patients (2.59%, 45 eyes) were diagnosed with advanced AMD. Exudative (wet) AMD (including macular disciform scars) was identified in nine subjects (10 eyes), which was 0.56% of those who completed the study and 21.4% of advanced AMD patients. Atrophic (dry) AMD was found in 33 cases (35 eyes), which was 2.04% of the final number of patients enrolled and 78.57% of advanced AMD patients.

Combined FFA and OCT examination provided the highest detection rate of AMD for early and intermediate-stage AMD patients, which was significantly better than the detection rates when the techniques were performed alone (all P<0.05; Tables I and II).

### Follow-up observations

**Figure 1:**
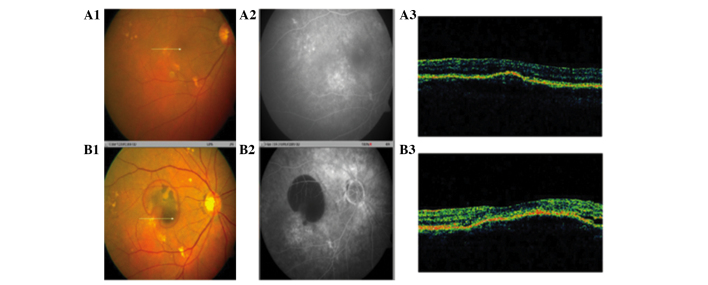
Representative fundus photography, FFA and OCT images of the exudative changes observed in AMD cases. (A1) Fundus examination of the right eye showed multiple drusens and pigmentary changes of the macula. (A2) FFA showed minimal leakage of a drusen with a diameter of 940 μm in the macula fovea. (A3) OCT revealed a medial signal of PED in the same place, and the area of PED shows shadow underlying the choroid, indicating fibrovascular PED. After 15 months of follow-up: (B1) Fundus examination showed a 3 PD area of edema presented in the former drusen area with retinal hemorrhage. (B2) FFA showed a 3 PD oval-shaped blocked fluorescent signal with a high leak from the lower temporal region. (B3) OCT revealed enlargement of the fibrovascular PED together with serous detachment of the nerve-fiber layer. FFA, fundus fluorescein angiography; OCT, optical coherence tomography; AMD, age-related macular degeneration; PED, pigment epithelium detachment; PD, pupillary distance.

**Figure 2:**
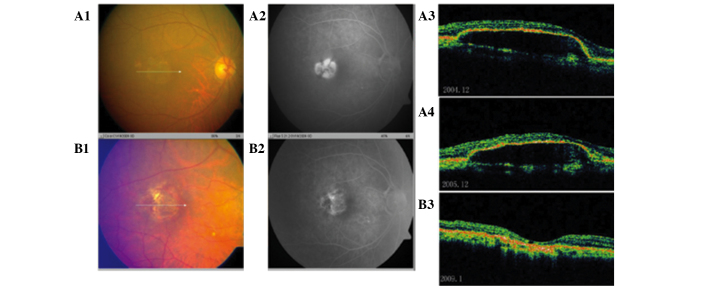
Representative fundus photography, FFA and OCT images of geographic atrophy changes in AMD cases. (A1) Fundus examination of the right eye revealed an oval-shaped 1 PD pigmentary detachment. (A2) FFA of the corresponding region showed a well-demarcated hyperfluorescent lesion with small sections of blocked fluorescent. (A3) OCT revealed a large quantity of subretinal serous fluid causing PED with atrophy of the nerve-fiber layer. After 12 months of follow-up: (A4) OCT revealed shrinkage of the former PED, and the local enhancement of reflective signals in the detachment cavity indicated absorption of the serous. After 49 months of follow-up: (B1) Fundus examination showed 2 PD geographic atrophy of the lesion. (B2) FFA revealed a mottled pattern of fluorescein staining. (B3) OCT showed the atrophy of nerve-fiber layer and the hyper-reflectivity of the underlying choroid from the former PED area. FFA, fundus fluorescein angiography; OCT, optical coherence tomography; AMD, age-related macular degeneration; PED, pigment epithelium detachment; PD, pupillary distance.

**Figure 3:**
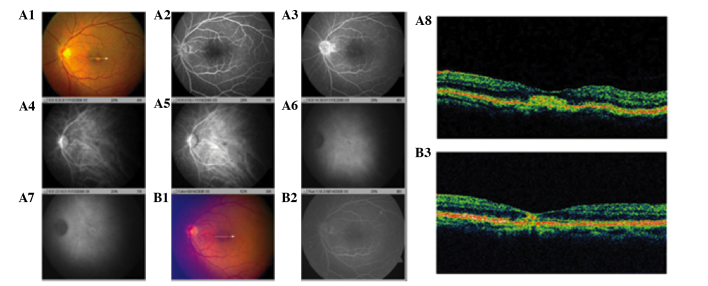
Fundus photography, FFA and OCT images from one patient whose calcific drusens were absorbed during the 33-month follow-up. (A1) Fundus examination of the left eye showed a 660-μm diameter grey-white lesion in the fovea. (A2) FFA revealed transmitted fluorescence surrounding the drusen in the early phase. (A3) Late phase FFA showed fluorescence staining of the drusen without leakage. (A4 and A5) Early phase ICG showed hypofluorescence of the drusen. (A6) ICG showed mild fluorescence staining of the drusen. (A7) Late phase ICG showed non-fluorescent leakage. (A8) OCT revealed an irregular hyper-reflective band in the RPE just under the former lesion. After 39 months of follow-up: (B1) Fundus examination revealed the disappearance of the drusen with the remaining depigmentation. (B2) FFA exhibited no evident abnormality. (B3) OCT scans showed a molted hyper-reflective band of the former lesion with discontinuity of the RPE reflective band and evident thinning of the nerve-fiber layer. FFA, fundus fluorescein angiography; OCT, optical coherence tomography; ICG, indocyanine green; RPE, retinal pigment epithelium..

**Figure 4:**
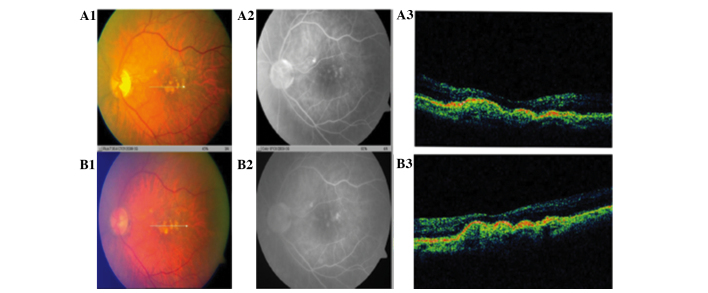
Representative fundus photography, FFA and OCT images of increased or fused drusens in AMD cases. (A1) Fundus examination of the left eye showed multiple confluent drusens in the fovea with a diameter of >1,200 μm. (A2) FFA exhibited late mild fluorescence staining of the drusens without fluorescence. (A3) OCT scans showed an irregular enhanced reflectivity band of the RPE in the drusen area with thinning of the nerve-fiber layer. After 46 months of follow-up: (B1) Fundus examination revealed further confluent drusens. (B2) FFA showed partial confluent drusens without fluorescence leakage. (B3) OCT revealed a more severe irregular reflection band of the RPE in the drusen area. FFA, fundus fluorescein angiography; OCT, optical coherence tomography; AMD, age-related macular degeneration; RPE, retinal pigment epithelium.

In total, 59 early and intermediate-stage AMD patients were followed-up for 18–54 months (mean follow-up time, 38.6±11.3 months) and all underwent complete ocular examinations during this time. Five eyes (3.62%) developed into geographic atrophy 56 months after the initial recruitment examination (mean duration, 43.6±9.3 months; Fig. 1). A total of four eyes (2.9%) developed into exudative AMD between 22 and 37 months following the initial examination (mean duration, 21.2±12.8 months; Fig. 2). Calcific drusens were absorbed in one eye (0.72%) 33 months following the initial recruitment examination (Fig. 3). In 40 eyes (56.5%), increased or fused drusens were observed between 44 and 60 months subsequent to the initial recruitment examination (mean duration, 47.6±10.5 months; Fig. 4).

## Discussion

As China becomes an aging society, the prevalence of age-related diseases has significantly increased. AMD is one of the leading causes of blindness and poor vision, resulting in an inability to work or live independently, which adds a heavy burden on the individual, the family and society. Since macular damage is irreversible, early detection and timely treatment are important in preventing the enlargement and coalescence of drusens and early CNV, thereby reducing or delaying visual impairment and improving the patients’ quality of life (9–12).

An epidemiological survey of AMD in Youyi Road Community, Baoshan District, Shanghai, China was performed between April 2005 and June 2005. Individuals with early and intermediate-stage AMD were selected to investigate the pathomorphological and functional variations of CNV in AMD patients in a Chinese population. The natural course of AMD was satisfactorily observed and recorded using OCT combined with FFA. FFA is useful for assessing the range of RPE damage, while OCT can quantify the thickness of the neurosensory retina and detect traces of subretinal fluid and early thinning of the neural cortex in atrophic AMD. In addition, OCT can be used to determine the degree of damage to the photoreceptors, Bruch’s membrane and the choriocapillary layer (CCL) (13).

The clinical staging criteria of the Age-Related Eye Disease Study do not include a clear definition of vision. It is increasingly recognized that patients with early and intermediate-stage AMD without macular involvement can usually maintain good eyesight. Furthermore, visual impairment is usually gradual and severity varies with the lesion site. As a result, classification of AMD is less meaningful if stages of its pathogenesis are based on vision. Thus, we hypothesize that it is more practical in clinical practice to base the severity of AMD on the fundus phenotype as viewed by OCT and FFA, rather than declined vision.

OCT and FFA were combined to diagnose and follow-up patients with early and intermediate-stage AMD, focusing on the organic and functional changes observed in the macular area of the photoreceptors, RPE and CCL. Detection via combined OCT and FFA provides a more accurate and objective assessment of AMD damage. Fundus photography revealed that there were two eyes (2.9%) that developed exudative changes, including unclear drusen borders and proliferating pigments, among the 59 patients (70 eyes) with early and intermediate-stage AMD that were followed-up for 18–54 months. The aforementioned cases presented with central blocked fluorescence in the center of the drusens, fuzzy weak fluorescence around the drusens and mild enhanced fluorescence in advanced stages of FFA imaging. As exudative changes advanced, the original drusens became obscure and large areas of subretinal or subpigmental hemorrhages appeared in the fundus images. In addition, the bleeding site was blocked, fluorescein leakage had occurred due to neovascularization and fluorescence accumulation in cavities of pigment epithelial detachment and neurosensory retinal detachment were observed through FFA. OCT showed wide range of dome bands within the RPE/CCL area (14,15). When geographic atrophy occurred, RPE serous detachment in the macular area, over a range of one pupillary distance, was observed in the fundus images, FFA and OCT scans. During the follow-up, RPE detachment was gradually absorbed, the pigments were deposited and eventually the photoreceptors, RPE and choroidal capillaries became thinner.

In conclusion, FFA is a valuable tool for making a definitive diagnosis of AMD, however, the invasiveness of the procedure limits the usage on a single patient. OCT is a noninvasive test that can provide an *in vivo*, objective and precise measurement of macular and retinal nerve fiber layer thickness. OCT has become popular and is used widely in clinical practice worldwide. The advantages and characteristics of this technique have been thoroughly described previously (16–18). OCT enables ophthalmologists to diagnose retinal diseases by detecting small changes in retinal and macular morphology, including the thickness and volume. As a result, OCT is an easier and more comfortable method to follow-up patients with early and intermediate-stage AMD, and the facilitation of OCT in the diagnosis of retinal diseases contributes to a positive significance in clinical practice.

## Figures and Tables

**Table I:** Comparison of three diagnostic methods for early and intermediate-stage AMD.

Diagnostic method	Cases, n	Detected cases, n	Detection rate, %
Fundus examination	1619	39	2.41
FFA	1325	48	3.62
OCT	1619	62	3.83
Combined FFA and OCT	1325	59	4.45

AMD, age-related macular degeneration; FFA, fundus fluorescein angiography; OCT, optical coherence tomography.

**Table II:** Comparison of three diagnostic methods for advanced-stage AMD.

Diagnostic method	Case, n	Detected cases, n	Detection rate, %
Fundus examination	1619	50	3.09
FFA	1325	36	2.72
OCT	1619	42	2.59
Combined FFA and OCT	1325	42	3.17

AMD, age-related macular degeneration; FFA, fundus fluorescein angiography; OCT, optical coherence tomography.
